# Selection and Presentation of Imaging Figures in the Medical Literature

**DOI:** 10.1371/journal.pone.0010888

**Published:** 2010-05-28

**Authors:** George C. M. Siontis, Nikolaos A. Patsopoulos, Antonios P. Vlahos, John P. A. Ioannidis

**Affiliations:** 1 Clinical Trials and Evidence-Based Medicine Unit, Department of Hygiene and Epidemiology, University of Ioannina School of Medicine, Ioannina, Greece; 2 Department of Pediatrics, University of Ioannina School of Medicine, Ioannina, Greece; 3 Biomedical Research Institute, Foundation for Research and Technology-Hellas, Ioannina, Greece; 4 Department of Medicine, Institute for Clinical Research and Health Policy Studies, Tufts University School of Medicine, Boston, Massachusetts, United States of America; 5 Department of Epidemiology, Harvard School of Public Health, Boston, Massachusetts, United States of America; University Paris 7, France

## Abstract

**Background:**

Images are important for conveying information, but there is no empirical evidence on whether imaging figures are properly selected and presented in the published medical literature. We therefore evaluated the selection and presentation of radiological imaging figures in major medical journals.

**Methodology/Principal Findings:**

We analyzed articles published in 2005 in 12 major general and specialty medical journals that had radiological imaging figures. For each figure, we recorded information on selection, study population, provision of quantitative measurements, color scales and contrast use. Overall, 417 images from 212 articles were analyzed. Any comment/hint on image selection was made in 44 (11%) images (range 0–50% across the 12 journals) and another 37 (9%) (range 0–60%) showed both a normal and abnormal appearance. In 108 images (26%) (range 0–43%) it was unclear whether the image came from the presented study population. Eighty-three images (20%) (range 0–60%) had any quantitative or ordered categorical value on a measure of interest. Information on the distribution of the measure of interest in the study population was given in 59 cases. For 43 images (range 0–40%), a quantitative measurement was provided for the depicted case and the distribution of values in the study population was also available; in those 43 cases there was no over-representation of extreme than average cases (p = 0.37).

**Significance:**

The selection and presentation of images in the medical literature is often insufficiently documented; quantitative data are sparse and difficult to place in context.

## Introduction

Images convey important information for both academic and clinical purposes in the radiological literature and beyond. However, there is no formal written guidance to our knowledge on how to select and present images [1]. It would be useful to understand if and how authors provide representative images and adequate information on them to support their findings. Selection and presentation of figures could have important implications for the interpretation and application of information from figures in medical practice.

Here, we evaluated in a relatively large sample of medical articles carrying radiological images, how the imaging figures are reported, whether the authors mention how and why they selected them, whether quantitative information is furnished regarding the published images and the study population they are derived from, and whether images are representative of the overall population or extreme cases are preferentially depicted.

## Methods

### Selection of studies

We screened all the issues of 3 major general (*JAMA, Lancet, NEJM*) and 9 major specialty (*American Journal of Obstetrics & Gynecology, American Journal of Psychiatry, American Journal of Respiratory & Critical Care Medicine, Arthritis & Rheumatism, Circulation, Gastroenterology, Neurology, Pediatrics, Radiology*) medical journals published in 2005. The 9 specialty journals are those that receive the highest annual citations in the specialties of Radiology, Neurology, Psychiatry, Rheumatology, Cardiology, Respiratory and Critical Care Medicine, Gastroenterology, Pediatrics, and Obstetrics and Gynecology, according to Thomson Journal Citation Reports [2]. We decided to search for eligible articles including images by searching all the articles of a specific year one-by-one by hand, so as to maximize sensitivity for finding the eligible articles. We selected original studies of any design on humans that included imaging figures on any part or anatomical system of the human body, derived by any imaging technique. Reviews without original data, single case reports (including single-family reports) and non-human studies were excluded. Moreover, we excluded endoscopic images, images from tissues or cadaveric specimens, plain human body photographs, and images of tissues or cells.

Some journals publish numerous imaging studies, while others publish far fewer such studies. To avoid the evaluated sample being overwhelmed by the first category, when more than 30 eligible articles were identified in a journal, we randomly selected 30 articles (by using the function “sampsi” in STATA 10.0) for further evaluation.

Eligibility assessment was performed by three independent evaluators. Discrepancies were further resolved by consensus and arbitration by a fourth investigator. All three investigators who performed data extraction are physicians, and one of them is an expert on cardiovascular imaging and ultrasound, serving as faculty and attending at a university hospital and directing an ultrasound service. The arbitrating investigator is a physician with professor appointments at both epidemiology and clinical departments.

### Data extraction

We scrutinized each figure along with its legend, and all the relevant text or other material that was presented in an article, including even any online supplements. Due to the great variety in terms of scope, subject and presentation across the included studies in our evaluation, we focused only on image aspects that are common and nonspecific.

For each figure, we recorded the imaging technique under investigation and the sample size of the included population (reference study population). Imaging techniques were categorized in six main subgroups: radiography (chest X-ray, esophagogram, mammogram, fluoroscopy, invasive angiography etc); ultrasonography (US); computed tomography (CT); magnetic resonance imaging (MRI); other (conventional nuclear medicine examinations, single photon emission computed tomography (SPECT), positron emission tomography (PET), optical coherence tomography); and combination of the previous techniques (images of different techniques in the same figure on the same subject). For each article we recorded whether the primary objective was to introduce/evaluate the characteristics of an imaging technique or apply an established technique; and whether the imaging was also an intervention.

For each eligible figure, we scrutinized the legend and text of the article and recorded verbatim the authors' comments, if any, about the selection of the specific image. In particular, we recorded whether the comments suggested that the selected case was considered to be representative without clarifying whether this means average or extreme/clear-cut; an average case; an extreme case; or a normal case.

If more than one imaging figure existed in a study, each figure was accounted for separately. Each eligible figure was examined on whether it refers to a subject(s) of the population under investigation (study population) or not. When no information was provided whether the imaged subject(s) belonged to the study population or not, we recorded this as “unclear”.

For each imaging figure we recorded whether any quantitative (e.g. “ejection fraction 40%”, “stenosis 80%”), or at least ordered categorical (e.g. grade 2) information was provided for the main item/measure of interest in the figure; or only non-quantitative information was given. The figure legend and the corresponding text and tables were screened. When only a reference scale was provided in the figure, but no specific number was already measured and reported, we did not count this as provision of quantitative information. When many items/measures were given per figure we gave preference to select the quantitative over ordered categorical and over non-quantitative. We then recorded also the specific quantitative value(s) presented in the image.

For studies that reported on quantitative or ordered categorical measures, we also recorded whether the distribution of the values of the study population for the measure of interest presented in the image was provided. Information on the distribution of values could be provided through presentation of measurement(s) per subject (individual-level data), or presentation of mean ± standard deviation (SD), mean or median and interquartile range (IQR) or other information that would help understand the distribution of the values. In 13 images where more than one type of quantitative measure were shown in the same image, we preferred to keep the measure where the distribution of values in the study population was also available (n = 9 images); while if the distribution was given for no measure (n = 1 image) or for more than one (n = 3 images), we selected the measure mentioned first in the results.

Furthermore, we examined the reporting of color signals, whether quantitative color scales were provided and whether these were numbered. For imaging techniques where contrast is possible to use, we recorded how many images came from articles that did not state whether contrast was used or not and this was also not clarified in the specific imaging figure; how many imaging figures came from articles that stated in the Methods or elsewhere that contrast was used in all the imaging; and how many images came from articles that stated that contrast was used in some of the imaging. In the latter category, we recorded how many figures stated that contrast had been used and how many stated that contrast had not been used.

Two evaluators independently extracted the data and a third independent evaluator was also added for the quantitative analyses. Another evaluator arbitrated on discrepancies. The data extraction form is presented in **Table S1**.

### Analyses

We described and summarized what comments had been made (if any) by the investigators on the selection of the image, and calculated the proportion of imaging figures where it was clear that the images came from the study population, where any quantitative or ordered categorical measure of interest was shown, and where the distribution of values in the study population was given for the measure of interest. The percentage of images satisfying each of these qualitative criteria was compared across all journals and for the comparison of *Radiology* versus other non-radiology journals, using an exact test.

We also evaluated in how many imaging figures, the distribution of values was available in the study population and also the quantitative value was provided also for the shown image(s). In these cases, we placed the presented image(s) against the respective distribution of the study population where it belonged to, by estimating the standardized value for the presented image(s). For example, a standardized value of 0.0 means that the measure of interest in the shown image is the average of the study population; a standardized value of 1.0 means that the measure of interest in the shown image is 1.0 standard deviation higher than the average (i.e. higher than approximately 84% of the values of the study population); and a standardized value of 2.0 means that the measure of interest in the shown image is 2.0 standard deviations higher than the average (i.e. higher than approximately 97.5% of the values of the study population). When individual-level data were not provided, information on mean (and SD) and median (and IQR) was used considering the study population to be normally distributed, unless otherwise stated in the article. When an image showed several different cases, we counted these separately, but while when several measurements of the same case under the same conditions were provided, we only kept the average of these replicates. We then used the Kolmogorov-Smirnov test to evaluate the null hypothesis that the standardized values of the shown images are drawn from a normal distribution i.e. there is no preference (or avoidance) for showing extreme cases from the tails of the distribution.

Analyses were conducted in SPSS 15.0 (SPSS Inc., Chicago, IL), STATA 10.0 (STATA Corp) and StatXact 3.0 (Cytel Corp., Boston, MA). P-values are two-tailed.

## Results

### Eligible articles

**Figure 1:**
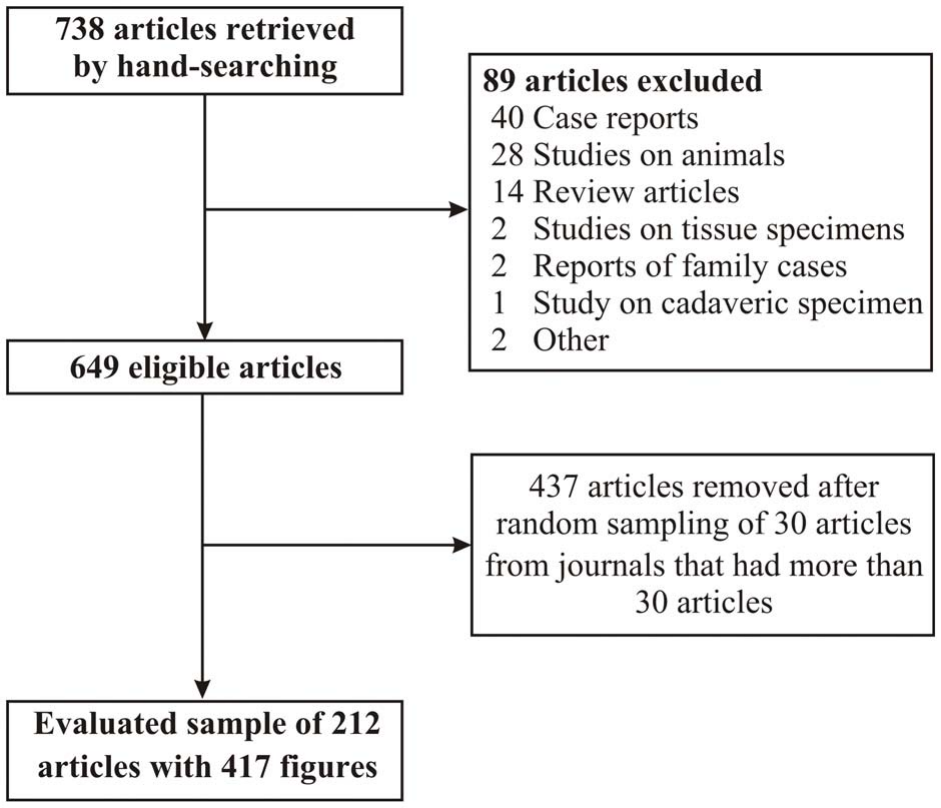
Flow chart for retrieved, eligible and analyzed studies.

A total of 738 potentially eligible articles, which contained at least one image figure, were identified. Eighty nine articles were excluded (**Figure 1**). Overall, 649 studies that were published in the 12 journals fulfilled our inclusion criteria (**Table 1**). The large majority of articles appeared in leading specialty rather than general journals (636/649). Moreover, more than half of the eligible articles (52%) were published in Radiology, and many were also published in Circulation (77 articles) and Neurology (115 articles). After selecting randomly only 30 articles from each of these 3 journals, we created the final sample of 212 eligible articles (**References S1**) that were analyzed in depth (**Table 1**). The number of patients in the study population(s) of the included studies (n = 212) ranged from 4 to 12 672. Seventy-eight of the 212 articles (37%) had as their primary objective to introduce/evaluate an imaging technique. Nine articles (4%) used at least one interventional imaging procedure.

**Table 1:** Eligible and analyzed articles and images.

Journal	Articles (analyzed)	Sample size median (IQR)	Images (panels)	Images with ≥2 panels	Type of images					
					Rad	US	CT	MRI	Other	Combo
**Am J Obst & Gyn**	27 (27)	76 (48–302)	65 (137)	30	0	55	0	10	0	0
**Am J Psych**	30 (30)	29 (24–47)	50 (282)	44	0	0	0	38	11	1
**Am J Resp & Crit Care Med**	8 (8)	33 (21–106)	14 (51)	9	1	2	3	6	2	0
**Arthr & Rheum**	24 (24)	57 (31–192)	37 (114)	28	6	1	0	29	0	1
**Circulation**	77 (30)	41 (26–60)	58 (191)	41	15	16	8	12	4	3
**Gastroenterology**	6 (6)	13 (8–52)	16 (62)	14	0	3	0	5	4	4
**JAMA**	3 (3)	456 (103–492)	6 (12)	4	1	0	4	1	0	0
**Lancet**	4 (4)	93 (64–361)	5 (20)	5	0	1	2	1	1	0
**NEJM**	6 (6)	54 (25–76)	6 (14)	3	0	1	0	2	1	2
**Neurology**	115 (30)	40 (20–83)	51 (314)	48	0	1	5	37	2	6
**Pediatrics**	14 (14)	84 (51–136)	22 (45)	15	2	6	2	12	0	0
**Radiology**	335 (30)	40 (19–65)	87 (265)	67	7	7	31	31	0	11
**Total**	**649 (212)**		**417 (1507)**	**308**	**32 (8%)**	**93 (22%)**	**55 (13%)**	**184 (44%)**	**25 (6%)**	**28 (7%)**

Am J Obst & Gyn: American Journal of Obstetrics & Gynecology; Am J Psych: The American Journal of Psychiatry; Am J Resp & Crit Care Med: American Journal of Respiratory and Critical Care Medicine; Arthr & Rheum: Arthritis & Rheumatism; IQR: Interquartile range; Rad: Radiography; US: Ultrasonography; CT: Computed Tomography; MRI: Magnetic Resonance Imaging; Combo: Combination.

### Eligible imaging figures evaluated

The imaging figures per article ranged from 1 to 7 (**Table 1**) for a total of 417 imaging figures analyzed. Conventional radiographs or any other diagnostic study based on fluoroscopic techniques were uncommon and accounted for only 8% of the 417 imaging figures. Almost half of the figures (44%) pertained to MRI (range 15–84% across the 12 included journals). US (22%) (range 0–85%) and CT (13%) (range 0–40%) were also common.

### Qualitative statements about selection

Forty-four imaging figures (11%) (range 0–50% across journals) made at least some more specific comment or hint on whether they were showing representative, average, extreme, or normal examples (such comments appear verbatim in **Table S2**). Most of these specific comments suggested a representative selection without clarifying whether this meant an average, extreme or normal case (n = 22) (the terms used were “representative” [n = 9 images], “typical” [n = 10 images], “sample case(s)” [n = 3 images]; we do not count here images referred simply as “examples” or “for illustration” without any further characterization). Only for 2 images, the language was more specifically describing an average case selection and in another 9 images, the comments suggested an extreme case selection (“far laterally”, “only identified in…”, “one major anomaly”, “outlier”, “the strongest”, “extensive”, “large” (n = 2 images), “ selectively shows”). Finally, only 11 (2.6%) images clearly stated that they were showing a normal case, focusing on the fact that this is the normal appearance (statements such as “normal”, “healthy volunteer” and “healthy subjects” were used; three of them also used the term “representative” and one also used the term “typical”).

In another 37 (9%) (range 0–60% across journals) images both a “normal” and one or more “abnormal” case were presented for comparison, but there was no comment/hint about the selection/representation of the shown abnormal cases. This included 30 figures with two panels (or more) each showing subjects with “normal” vs. “abnormal” features, 3 figures with two panels each on pre-intervention/abnormal vs. post-intervention/normal on the same patient and 4 figures with comparison of normal vs. abnormal regions of the brain on the same panel (all of them on fMRI results).

### Qualitative evaluation of reporting of images

In 108 (26%) (range 0–43% across journals) of the imaging figures it was not possible to determine whether the image referred to one of the cases of the study population or not (**Table 2**). There was diversity across journals in the proportion of images where it was clear that the image was derived from the study population (58–100%, p<0.001 by exact test), with higher percentage for Radiology than for non-radiology journals (p = 0.002).

**Table 2:** Characteristics of the imaging figures.

Journal	Image stated to be derived from the study population			Type of measure presented in the image			Distribution of values in the study population^a^	
	No	Yes	Unclear	Quantitative	Ordered Categorical	Neither	Information not provided	Information provided
**Am J Obst & Gyn**	1†	35	29	10	0	55	1	9
**Am J Psych**	0	40	10	7	0	43	7	0
**Am J Resp & Crit Care Med**	0	9	5	0	0	14	0	0
**Arthr & Rheum**	0	22	15	2	5	30	3	4
**Circulation**	0	37	21	8	2	48	0	10
**Gastroenterology**	0	15	1	4	0	12	0	4
**JAMA**	0	5	1	0	0	6	0	0
**Lancet**	0	4	1	3	0	2	1	2
**NEJM**	0	6	0	0	1	5	0	1
**Neurology**	0	46	5	5	2	44	5	2
**Pediatrics**	0	13	9	1	0	21	1	0
**Radiology**	0	76	11	30	3	54	8	25
**Total**	**1 (0.2)**	**308 (73.9)**	**108 (25.9)**	**70 (16.8)**	**13 (3.1)**	**334 (80.1)**	**26 (31.3)**	**57 (68.7)**

Values in Total given as n (%).

Journal abbreviations as per Table 1.

a For those images where data on quantitative or ordered categorical measures where given in the image.

†image reproduced from a previous publication with explicit reference to that publication.

The authors provided any quantitative information on a measure of interest in 70 (17%) of the images (range 0–60% across journals), and another 13 (3%) had ordered categorical information (range 0–17%). There was diversity across journals in the proportion of images that included quantitative/ordered categorical information (p<0.001 by exact test) with higher percentage for Radiology than for non-radiology journals (p<0.001).

Any information on the distribution of the main measure of interest on the images was detected in 57 (69%) of the figures (range 0–100% across journals) where any quantitative or ordered categorical information was available, with significant diversity across journals (exact p<0.001) and a non-significantly higher proportion in Radiology than in non-radiology journals (76% vs. 64%, p = 0.34). Forty-nine of the above images were clearly from the study population.

For 106 images (25%) color signals were shown for various techniques (US, CT, MRI, PET, SPECT) (range 0–70% across journals) and 5 of these 106 were in Radiology. A quantitative scale on the color signal for a rough evaluation of the colors shown was provided in 48 of these images (3 of which in Radiology) (**Table 3**). The proportion of images that provided numbered color scales ranged from 0–80% across journals (exact p<0.001); there were very few such images in Radiology to allow a meaningful statistical comparison against other journals.

**Table 3:** Presentation of information on color scales and use of contrast.

Journal	Color scales for figures with color signals		Stated contrast use^a^		
	Provided numbered scale	No provided scale or scale unnumbered	In all the study population	In part of the study population	Not stated
**Am J Obst & Gyn**	9	2	1	0	9
**Am J Psych**	10	25	0	0	39
**Am J Resp & Crit Care Med**	3	2	0	0	9
**Arthr & Rheum**	3	0	17	1	12
**Circulation**	6	4	30	0	7
**Gastroenterology**	0	8	0	0	9
**JAMA**	0	0	4	0	1
**Lancet**	1	1	0	0	3
**NEJM**	0	0	0	1	3
**Neurology**	13	14	5	3	40
**Pediatrics**	0	0	0	4	12
**Radiology**	3	2	58	0	19
**Total**	**48**	**58**	**115**	**9**	**163**

Journal abbreviations as per Table 1.

a For those images that were obtained with techniques where contrast may be used.

Overall we identified 287 figures (published in 145 articles) that pertained to an imaging technique where contrast may be used. In 163 figures (published in 93 articles) the authors did not make any statements regarding the use of contrast agent or not, whereas in 46 articles (including 115 figures) it was clearly stated that a contrast agent was used in all cases of the study population. For 6 articles (including 9 figures) it was stated that contrast was used in some of the presented cases and in 5 of the 9 figures the authors reported the use of a contrast agent for each specific figure either in the figure legend or the main text. The proportion of images with information on contrast use varied from 0–100% across journals (exact p<0.001) and it was higher in Radiology than other journals (p<0.001) (**Table 3**).

### Representation: quantitative evaluation

**Figure 2:**
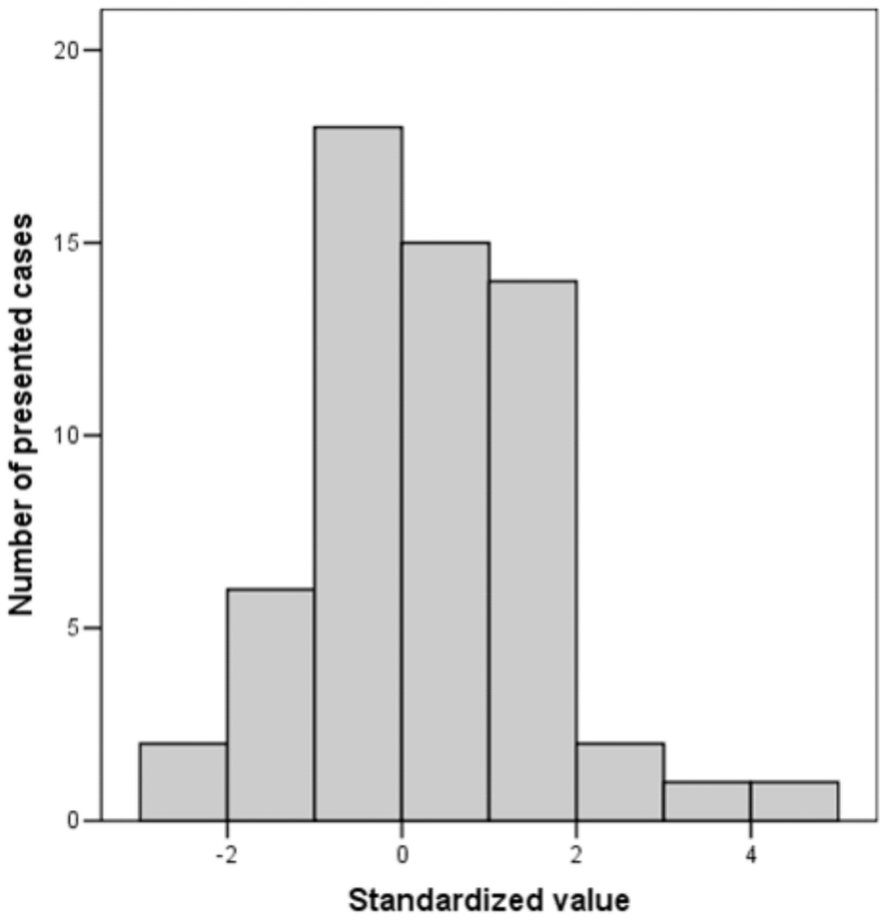
Distribution of standardized values for images where the value of the measure of interest was presented and it could be translated to a standardized value against the values in the respective study population.

Forty three images (showing 59 different cases) (range 0–40% across journals) had quantitative information that could be placed against the respective study distribution (**Figure 2**; for details see **Table S3**). The Kolmogorov-Smirnov test showed no significant deviation from normality (p = 0.37) and there was no clear evidence for heavy tails, i.e. preference for showing extreme rather than average cases.

### Incomplete imaging reporting: illustrative examples

A couple of illustrative examples that highlight incomplete reporting issues and potential lack of useful information are presented below:

#### Example #1

In their Figure 1, Hermoye et al. [3] show a figure with two side-by-side panels, one of the manual and the other of the semiautomatic segmentation method. The two panels look almost identical. One thus gets the impression that the two methods give the same results. The legend claims that this is a “representative” picture. However, the text of the paper implies that there are limits in the agreement between the two methods, thus apparently in other cases the segmentation may not be so similar and ideal as the presented figure implies. It might have been informative to also have a figure where the agreement of the two methods is suboptimal. Moreover, we do not have an exact appreciation of where the presented picture stands in the spectrum of the study population, despite the use of the general term “representative” in the legend.

#### Example #2

In their Figure 1, Tack et al. [4] provide images of a CT pulmonary angiography that displays a filling defect in different doses/settings. The method produced different levels of agreement in different segments, but the figure gives the impression that a filling defect is absolutely the same no matter what the settings are.

## Discussion

We have evaluated the selection, reporting and representation of 417 radiological imaging figures in 212 articles published in high-impact journals. In most, no comment or hint was made by the authors regarding the selection and representativeness of the images. Sometimes it was unclear whether the image was derived from the population of the study or not. Few images gave specific values for quantitative or at least categorical measurements for the depicted cases and information on the distribution of the major measure of interest in the study population was available in only two thirds of these images. Informative color scales were used in the minority of color images and many figures did not clarify whether contrast medium had been used or not. When quantitative information was available both for the depicted images and the study population, there was no evidence for selective presentation of extreme cases, but eventually such data were available for fewer than 1 in 8 images.

Our findings indicate the lack of standardized reporting of published images in the medical literature. This adds upon the existing evidence for suboptimal reporting of other aspects of the design and conduct of diagnostic studies [5]–[9], since most images pertain to diagnostic tests. Essential aspects that may often be necessary for the proper understanding and interpretation of an image from the majority of the readers are often missing or unclear. Quantitative data are sparse and appreciating whether the depicted case is an average case or something extreme is often difficult. In the small minority where quantitative information was provided for both the specific depicted image and the study population, we found no bias in favor of showing extreme cases, but this was a small sample of the images with the most meticulous quantitative reporting. It is unclear if this would apply also to the majority of images where this information was missing. Readers would wish to know whether the depicted images are representative of average or extreme examples. A contrast of normal versus abnormal features and the clarification of the use of a contrast agent in the specific image/technique would also be useful especially in new or complex imaging techniques, but both are also uncommonly used.

We have not addressed issues of image manipulation that have raised concerns in the basic biomedical sciences [10]–[12]. There is no evidence on whether image manipulation may be an issue also in clinical medicine, but unfortunately this is not possible to decipher easily from examination of printed radiological images. However, insufficient information may also diminish the value of the presented images and may also lead to misleading inferences among readers of this literature, even if no images are manipulated. Moreover, we focused in our analysis on imaging figures, but other types of figures are also important to present in clinical journals and Schriger et al. [13] have found relatively poor quality and possible misleading presentation of figures from submitted randomized trials.

There can often be an understandable tension between presenting an image, which is representative of a case study and one which is perhaps, less representative, but more instructive. Transparent reporting of the selection process does not mean that one should enforce what specific images authors should present. Simply, it would be useful to know whether the selection was based on the picture being representative or based on its instructive potential and special, perhaps atypical or even extreme features. Quantitative documentation and provision of further qualitative or more specific information may help in this regard, independently of the main purpose of the article.

Our study has limitations. First, we only examined 12 journals. However, the selected journals have high impact on clinical research and practice and it is unlikely that selection and reporting of images would be better in lower-impact journals. Second, we may have missed or misclassified information regarding images of highly specialized imaging techniques where only field specialists could properly evaluate them. Nonetheless, we used 3 data extractors and an arbitrator so as to minimize misconceptions. Third, we selected articles from a single year, therefore other studies may need to address whether there has been any improvement in selection and presentation of the figures over time. Fourth, we covered a substantial number of specialties, but it was impractical or even impossible to cover all specialties. Several other specialties, besides those that we examined, perform influential imaging studies. Nevertheless, it is unlikely that selection and presentation of images would be markedly different than what we observed across the considerable number of specialty journals we analyzed. If anything, some specialties may adopt imaging techniques and take them for granted using them without also showing any representative images. Fifth, we choose the highest-cited journals in each speciality and high citations do not necessarily mean also maximal clinical utility or maximal readability, but citation impact is easy to measure objectively, while readability and clinical impact may be more subjective. Sixth, quantitative analyses for the representation of the selected images against the study population distributions were based on more limited available data. Moreover, in some circumstances, the main inference from a figure may be simply whether a finding is present or not, with less importance for the exact measurement that defines its presence. However, even then, quantitative information can sometimes improve the accuracy and completeness of the presented information and can help place this finding in better context.

Selection of images is not a simple process. Images should help the authors and eventually the readers, in discussing their material. Carefully selected and presented images can enhance the quality of a paper [14]. This applies routinely to papers in imaging journals, but also to papers in other journals that do not specialize predominantly on imaging. Not surprisingly, Radiology, tended to have the best performance in the qualitative evaluations, but even there, we observed room for improvement.

In conclusion, some suggestions on reporting and presentation of images are summarized on **Table 4**. Such information would need to be complemented also with transparent and comprehensive reporting of other aspects of a study of diagnostic accuracy or any type of study where imaging is involved, for example, as specified by the STARD statement for diagnostic test evaluations [5], [6], [15]–[18].

**Table 4:** Items to be considered in selecting and presenting/reporting of images.

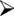 **Selection process of the published image:** Clarify if the intention to present an average case, an extreme case, or a selected case. If an extreme or a selected case, then clarify on what aspect.
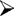 **Source of the image (study population or other):** Clarify if the image is derived from one of the cases under study in the specific paper (and if so, how selected, as above) or is an illustrative case not derived from the study population (and if so, explain why it is chosen).
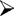 **Contrast of normal versus abnormal cases:** Consider providing side-by-side also a normal appearance and highlighting the key differences with the abnormal case(s) shown.
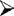 **Quantitative data on the presented case and the source population:** Consider providing quantitative numerical values on the main measurement(s) of interest, whenever pertinent, and provide information in the article on the distribution of the respective values across the study population, so that the specific presented image can be placed better in context.
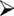 ** Color signals:** When color signals are shown, present an appropriate color scale and number the scale.
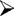 **Contrast specification:** Clarify whether contrast is used in the study in all or some images, and if so provide sufficient detail to allow understanding whether contrast has been used in each specific image.
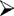 ** Other specific items:** Consider what other items may be important to convey that may be specific to the technique or image shown.

## Supporting Information

Table S1 A standardized instrument for evaluation of radiological images.
(0.05 MB PDF)

Table S2 Verbatim comments on selection/representation for shown cases.
(0.08 MB PDF)

Table S3 Quantitative information on measures of interest and derived percentiles.
(0.10 MB PDF)

References S1 List of included articles.
(0.12 MB PDF)
