# Supplementary material for: Selection and Presentation of Imaging Figures in the Medical Literature
Source: PLoS One. 2010 May 28;5(5):e10888. doi: 10.1371/journal.pone.0010888 (PMC2878319; doi:10.1371/journal.pone.0010888)
Supplement: Table S1 — A standardized instrument for evaluation of radiological images. (0.05 MB PDF) [file pone.0010888.s001.pdf]

Table S1. A standardized instrument for evaluation of radiological images

1. Sample size of the "study group" in which the image is pertained to: ….
2. Number of imaging figures: ….
3. Number of figure in article / Number of panels in each figure: …. / ….

   …. / ….

4. Type of radiological imaging technique:

   Radiography

   Ultrasonography

   Computed tomography

   Magnetic resonance imaging

   Other

   Combination of imaging techniques

5. Image clearly stated to be derived from the study group:

   No

   Yes

   Unclear

6. Type of the main measure of interest presented in the image:

   Quantitative

      Value of the main measure of interest in the image: ….

      Distribution of the main measure of interest in the study group: ….

   Ordered categorical

   Neither

7. Color scales for images with color signals:

Yes

Provided of numbered scale: Yes / No

No provided or unnumbered scale: Yes / No

No

8. Stated of the use of a contrast agent (where applicable techniques):

In all the study population

In part of the study population

In the specific image

Not stated

9. Provision of normal versus abnormal case for contrast: Yes / No

10. Authors' comments for the selection of the specific image:

Average case

Extreme case

Selected case

Other: ….
