# Supplementary material for: Selection and Presentation of Imaging Figures in the Medical Literature
Source: PLoS One. 2010 May 28;5(5):e10888. doi: 10.1371/journal.pone.0010888 (PMC2878319; doi:10.1371/journal.pone.0010888)
Supplement: Table S2 — Verbatim comments on selection/representation for shown cases. (0.08 MB PDF) [file pone.0010888.s002.pdf]

Table S2. Verbatim comments on selection/representation for shown cases

Journal	Volume (Issue)	Page	Verbatim Comment on Selection/Representation
Am J Obstet Gynecol	192(1)	257	Under normal conditions the aortic arch crosses the left mainstem bronchus and descends left to the midline along the spine
Am J Obstet Gynecol	192(1)	323	Representative aspect of the uterus and placenta by 3D sonography
Am J Obstet Gynecol	192(1)	323	Representative uterine Doppler
Am J Obstet Gynecol	192(1)	323	Representative power Doppler sonographs of the uterus 5 hours after delivery.
Am J Obstet Gynecol	192(2)	535	whereas most of the other anatomic regions had intermediate scores (3-4) (Table I, Figures 2 to 5)
Am J Obstet Gynecol	192(2)	648	Figure 1 shows typical, central sagittal slice images through the uterus at various time points over the menstrual cycle…Typical central sagittal slice through uterus at different phases of the normal menstrual cycle
Am J Obstet Gynecol	192(4)	1060	Typical appearance of transvaginal ultrasound measurement (2.23 cm) of a T-shaped cervix in the No Funnel group
Am J Obstet Gynecol	192(4)	1060	Typical appearance of transvaginal ultrasound measurements (1.22 cm = width of funnel, 1.09 cm

			= depth of funnel, 2.32 cm = distal closed cervical length, 3.41 cm = total cervical length) of a Y-shaped cervix in the Funnel group
Am J Obstet Gynecol	192(6)	1983	In 1 case, the catheter tip was observed to be far laterally in the region of the uterine cornua
Am J Obstet Gynecol	193(10)	1561	A normal profile of a fetus
Am J Obstet Gynecol	193(9)	1253	Ascending aorta and main pulmonary artery were visualized in 152 cases (95%), although the branch pulmonary arteries were only identified in 60 (37%) (Figure 2)
Am J Obstet Gynecol	193(9)	1260	One major anomaly, an open neural tube defect with meningocele, was detected at 13 weeks' gestation
Am J Obstet Gynecol	193(9)	762	Figure 1 illustrates representative ultrasound images of the anterior uterine wall of a woman in the P-CTR (Figure 1A) and PPROM (Figure 1B) groups.
Am J Obstet Gynecol	193(9)	1260	Normal appearance of anatomy at 11 to 14 weeks' gestation.
Am J Obstet Gynecol	192(1)	74	Ultrasound visualization of movement of abdominal viscera (visceral slide) during inspiration in an adhesion-free volunteer…healthy

			volunteers to confirm the feasibility of the technique and streamline the process.
Am J Resp Crit Care Med	171(6)	1298	A representative comparison of changes in crosssectional area at midtonsillar level (level 2) during tidal breathing of a subject with OSAS
Am J Resp Crit Care Med	172(12)	1497	Typical deposition patterns for the three monodisperse aerosols are shown in Figures 1 and 2.
Am J Resp Crit Care Med	172(8)	488	these appearances are typical…313 (99.4%) scans: 283 (89.8%) as consistent with IPF (Figure 1) and 30 (9.5%) as inconsistent (Figure 2 and Table 1)
Am J Resp Crit Care Med	172(8)	488	these appearances are typical…313 (99.4%) scans: 283 (89.8%) as consistent with IPF (Figure 1) and 30 (9.5%) as inconsistent (Figure 2 and Table 1)
Am J Psychiatr	162(5)	983	A representative subject is shown in Figure 3
Arthritis Rheum	52(11)	3528	The dGEMRIC lateral: medial ratio is determined by a small area of cartilage on the medial condyle (arrows) of a knee that is an outlier in Figure 5.
Arthritis Rheum	52(8)	2355	Coronal T1-weighted (a) and gadolinium-enhanced T1-weighted (b) images through the distal interphalangeal joint of an asymptomatic normal control subject
Arthritis Rheum	52(7)	2033	Representative sagittal T1-weighted fat-suppressed

			3-dimensional magnetic resonance images illustrating cartilage defects
Circulation	112(18)	2821	Typical contrast-enhanced images obtained by MRI.
Circulation	112(19)	2921	DIVPG image from a representative example.
Circulation	112(19)	2921	representative examples from a healthy volunteer (A, C, E, and G) and a patient with DCM
Circulation	112(19)	2980	a typical original recording of laser Doppler detected flow velocity after treatment with placebo (Figure 4B) and sildenafil
Circulation	112(21)	3320	Typical echocardiographic appearance of advanced carcinoid heart disease
Circulation	112(9) supp	I-396	Figure 4 demonstrates representative patients
JAMA	293(20)	2471	Sample Case in Individual With Nonstenosed Coronary Artery: Matched Negative Readings
JAMA	293(20)	2471	Sample Cases in Individuals With Stenosed Coronary Arteries:Matched Positive Readings
JAMA	293(20)	2471	Sample Case in Individual With Stenosed Coronary Artery: False-Positive Reading
Neurology	64(3)	542	Voxel locations and a typical patient spectrum from the left thalamus
Neurology	64(7)	1175	As illustrated in figure 1, the strongest activation was seen in the anterior cingulate/rostral

			midcingulate cortex (Z score 5.11)
Neurology	64(7)	1227	MRI and MR angiography findings of representative patients are shown in figure 2.
Neurology	64(8)	1384	images of a representative pairing of patients with left and right temporal lobe variants…from the remaining 19 patients with LTLV, six were chosen to mirror the RTLV group as closely as possible
Neurology	65(10)	1612	Three-dimensional MRI representation of hypometabolic areas showing the significant differences between patients with (A) and without (B) DP compared to 10 healthy volunteers using SPM99
Pediatrics	116(3)	717	Extensive CBH (ie, diffuse hemorrhage involving both cerebellar hemispheres and the vermis) was seen in 3 patients (9%) (Fig 2)
Pediatrics	116(4)	1001	1 infant in the WBC group had a large parenchymal hemorrhage with midline shift; this infant died.
Pediatrics	116(4)	1001	Another infant, with a severe coagulopathy before trial entry, developed a large hemispheric subdural hemorrhage with midline shift during head cooling, which resolved spontaneously
Radiology	234(3)	860	Figure 4 selectively shows the additional areas that were detected with 3.0-T MR imaging alone

Radiology	237(1)	353	Anteroposterior MIP from lower extremity CT angiography shows normal bilateral popliteal artery trifurcations and calf arteries,
Radiology	237(1)	57	Typical examples of malignant lesions are shown in Figures 4 and Figures 5; examples of benign lesions are given in Figures 6–8.
Radiology	237(2)	584	Oblique (a) sagittal and (b) coronal US images of normal supraspinatus muscle belly.
