# Supplementary material for: Selection and Presentation of Imaging Figures in the Medical Literature
Source: PLoS One. 2010 May 28;5(5):e10888. doi: 10.1371/journal.pone.0010888 (PMC2878319; doi:10.1371/journal.pone.0010888)
Supplement: Table S3 — Quantitative information on measures of interest and derived percentiles. (0.10 MB PDF) [file pone.0010888.s003.pdf]

Table S3. Quantitative information on measures of interest and derived percentiles

Journal	Volume (Issue)	Page	Sample size	Measure on the Figure	Distribution of the measure	Description of the measure	Standardized measure (SDs)	Any qualitative selection statements or normal/abnormal pairs
Am J Obstet Gynecol	192(1)	323	30	0.48; 0.75	0.48 ±0.07; 0.75±0.05	Resistance index, units not mentioned in text	0.00; 0.00	"Representative uterine Doppler aspect 5 hours after delivery"; "Representative uterine Doppler aspect 6 days after delivery."
Am J Obstet Gynecol	192(4)	1060	164	2.23	1.9±0.4	Distance, in cm	0.83	"Typical appearance of transvaginal ultrasound measurement (2.23 cm) of a T-shaped cervix in the No Funnel group."
Am J Obstet Gynecol	192(4)	1060	164	2.32	1.8±0.5	Distance, in cm	1.04	"Typical appearance of transvaginal ultrasound measurements (1.22 cm = width of funnel, 1.09 cm = depth of funnel, 2.32 cm = distal closed cervical length, 3.41 cm = total cervical length) of a Y-shaped cervix in the Funnel group."
Am J Obstet Gynecol	193(12)	2159	84	17.2	13.6±3.8, range 3.3-22.7	Volume of muscle, in cm3	0.95	None
Am J Obstet Gynecol	193(8)	387	294	0.61	0.403, range (10th-90th) 0.204-0.602	Umbilical cord index, no specific units; there is a description of what it measures	1.50	None

Am J Obstet Gynecol	193(9)	762	76	12.0	10.1±0.63	Uterine anterior wall thickness, in mm	3.0	"Representative ultrasonographic image of the anterior wall of the uterus with myometrial thickness measurement…" and presentation of "normal/abnormal pair"
Arthritis Rheum	52(11)	3528	31	459; 489	354±64; 375±65	Delayed gadolinium-enhanced MRI of cartilage index, in msec	1.64; 1.75	"The dGEMRIC lateral: medial ratio is determined by a small area of cartilage on the medial condyle (arrows) of a knee that is an outlier in Figure 5."
Circulation	112(15)	2318	52	79	Median 231, IQR 15-736	Calcium score	-0.45	None
Circulation	112(18)	2821	26	1%-25%; 26%-75%	1%-100% (given proportion with 1-25%, 26-100%)	Percentage of scar per sector	-1.2; 0.0	"Typical contrast-enhanced images obtained by MRI."
Circulation	112(19)	2921	60	3	2.5±0.8	Pressure, in mm	0.63	"DIVPG image from a representative example."
Circulation	112(25)	3892	55	-8	-7.2±7.3	End-systolic strain increment, dimensionless	0.11	None
Circulation	112(6)	855	48	20	22.5±9.6	Thickness, in mm	-0.26	None
Circulation	112(6)	855	48	35	22.5±9.6, extreme >30	Thickness in mm	1.30	None

Circulation	112(9) Supp	I-437	20	216	165± 48	Volume, in ml	1.06	None
Gastroenterology	128(5)	1179	15 (10 patients + 5 healthy volunteers)	3.25	Median 1.25 range 0.75-3.5 in the 10 patients	Time, in sec	1.50*	Presentation of "normal/abnormal pair"
Gastroenterology	128(5)	1179	15	1.25	median 1.25 range 0.75-3.5	Time, in sec	0.00	None
Gastroenterology	128(5)	1199	93	2.6;<br>1.7	3.3±0.2 (SEM) range 10th-90th 1.6-5.1	Distance, in cm	-0.53;<br>-1.21	None
Gastroenterology	128(5)	1199	93	3.1	3.0±0.3	Distance, in cm	0.33	None
Lancet	365 (9469)	1484	50	680;<br>960	743±76;<br>810±113	Thickness, in µm	-0.83;<br>1.33	Presentation of "normal/abnormal pair"
Lancet	366 (9498)	1711	108	0.2;<br>0.84	0.20±0.06;<br>0.41±0.10	RARE MRI technique	0.00;<br>4.30	Presentation of "normal/abnormal pair"
Neurology	64(8)	1354	20	30;<br>39;<br>45	40.1±17.6;<br>44.9±18.6;<br>48.3±19.1	Flow velocity, usually cm/sec not mentioned specifically	-0.57;<br>-0.32;<br>-0.17	None
Neurology	64(8)	1384	78 (58	mean of L-	L-3.27(-2.36to-4.15)	Volume z-scores	0.6; 0.4*	"Images of a representative pairing of patients with left and right temporal lobe

			patients)	3,53, R-3,47	R-3.29(-2.,67to-3.58) (values on 25 (6 R, 19 L) of 58 patients)			variants…from the remaining 19 patients with LTLV, six were chosen to mirror the RTLV group as closely as possible”
Radiology	234(2)	460	195	9	Range 9-20, mean 14 in the 81 patients with nodules	Diameter in mm	-2.25*	None
Radiology	234(2)	460	195	20	Range 9-20, mean 14	Diameter in mm	2.6*	None
Radiology	234(2)	460	195	10	Range 9-11, mean 10	Diameter in mm	0.00*	None
Radiology	235(2)	530	147	3	Range 1-3, 1.9±0.4 median 2	Diameter in mm	2.75	None
Radiology	235(2)	530	147	2	Range 1-3, 1.9±0.4 median 2	Diameter in mm	0.25	None
Radiology	235(2)	530	147	1.5	Range 1-3, 1.9±0.4 median 2	Diameter in mm	-1.00	None
Radiology	235(2)	530	147	1.5	Range 1-3, 2.0±0.6	Diameter in mm	-0.83	None
Radiology	235(3)	927	25	2	Range 2-4	Time difference, in sec	-1.7*	None

Radiology	236(2)	465	55	8.3	Mean 6.3 (range 0.39-28) in the n=35 patients with malignant lesions	Size, in cm^3	0.19*	None
Radiology	236(2)	465	55	0.39	Mean 6.3 (range 0.39-28)	Size, in cm^3	-2.09*	None
Radiology	236(2)	716	23	1.5	2.6 (range 1-4.6)	Tumor diameter, in cm	-1.18*	None
Radiology	236(2)	716	23	2.4	2.6 (range 1-4.6)	Tumor diameter, in cm	-0.21*	None
Radiology	236(2)	716	23	2.2; 1.5	2.6 (range 1-4.6)	Tumor diameter, in cm	-0.42; -1.18*	None
Radiology	236(2)	716	23	4.0; 4.2	2.6 (range 1-4.6)	Tumor diameter, in cm	1.20; 1.37*	None
Radiology	236(2)	716	23	1.8	2.6 (range 1-4.6)	Tumor diameter, in cm	-0.86*	None
Radiology	236(3)	810	51	67; 61	74±138; 77±138	Calcium score	-0.05; -0.12	None
Radiology	237(1)	57	65	149.9;	122±26.8;	Maximum & average	1.04;	"Typical examples of malignant lesions are shown in Figures 4 and Figures 5;

				121.1	88±24.5	Hb concentration, in µmol/L	1.35	examples of benign lesions are given in Figures 6–8.”
Radiology	237(1)	57	65	102.1; 68.7	122±26.8; 88±24.5	Maximum & average Hb concentration, µmol/L	-0.74; -0.78	“Typical examples of malignant lesions are shown in Figures 4 and Figures 5; examples of benign lesions are given in Figures 6–8.”
Radiology	237(1)	57	65	24.2	55±24.8	Maximum Hb concentration, in µmol/L	-1.24	“Typical examples of malignant lesions are shown in Figures 4 and Figures 5; examples of benign lesions are given in Figures 6–8.”
Radiology	237(1)	57	65	38.2; 26.3	55±24.8; 38±17.4	Maximum & average Hb concentration, in µmol/L	-0.68; -0.67	“Typical examples of malignant lesions are shown in Figures 4 and Figures 5; examples of benign lesions are given in Figures 6–8.”
Radiology	237(1)	57	65	94; 62.8	55±24.8; 38±17.4	Maximum & average Hb concentration, in µmol/L	1.57; 1.43	“Typical examples of malignant lesions are shown in Figures 4 and Figures 5; examples of benign lesions are given in Figures 6–8.”

When only the range is given for the distribution in the study population and the shown case has the maximum or minimal value, we assign to this the percentile 100-100/N or 100/N, respectively, where N is the sample size of the study population; when the mean m and the range is given, then the percentiles are assigned to the maximum and minimum, translated to z-scores, and then the observed values

are given z-scores proportional to the proportion of the distance that they lie between the mean and the maximum or minimum (depending on whether they are higher than the mean or lower than the mean, respectively).
