# Supplementary material for: Selection and Presentation of Imaging Figures in the Medical Literature
Source: PLoS One. 2010 May 28;5(5):e10888. doi: 10.1371/journal.pone.0010888 (PMC2878319; doi:10.1371/journal.pone.0010888)
Supplement: References S1 — List of included articles. (0.12 MB PDF) [file pone.0010888.s004.pdf]

References S1. List of included articles

S1. Chaoui R, Heling KS, Sarioglu N, Schwabe M, Dankof A, Bollmann R. Aberrant right subclavian artery as a new cardiac sign in second- and third-trimester fetuses with Down syndrome. Am J Obstet Gynecol 2005;192(1):257-263.

S2. Schaaps JP, Tsatsaris V, Goffin F, et al. Shunting the intervillous space: new concepts in human uteroplacental vascularization. Am J Obstet Gynecol 2005;192(1):323-332.

S3. Tu FF, Lamvu GM, Hartmann KE, Steege JF. Preoperative ultrasound to predict infraumbilical adhesions: a study of diagnostic accuracy. Am J Obstet Gynecol 2005;192(1):74-79.

S4. Darai E, Thomassin I, Barranger E, Detchev R, Cortez A, Houry S, Bazot M. Feasibility and clinical outcome of laparoscopic colorectal resection for endometriosis. Am J Obstet Gynecol 2005;192(2):394-400.

S5. Fouron JC, Gosselin J, Raboisson MJ, Lamoureux J, Tison CA, Fouron C, Hudon L. The relationship between an aortic isthmus blood flow velocity index and the postnatal neurodevelopmental status of fetuses with placental circulatory insufficiency. Am J Obstet Gynecol 2005;192(2):497-503.

S6. von Kaisenberg CS, Kuhling-von Kaisenberg H, Fritzer E, Schemm S, Meinhold-Heerlein I, Jonat W. Fetal transabdominal anatomy scanning using standard views at 11 to 14 weeks' gestation. Am J Obstet Gynecol 2005;192(2):535-542.

S7. Hoad CL, Raine-Fenning NJ, Fulford J, Campbell BK, Johnson IR, Gowland PA. Uterine tissue development in healthy women during the normal menstrual cycle and investigations with magnetic resonance imaging. Am J Obstet Gynecol 2005;192(2):648-654.

S8. Alcázar JL, Castillo G. Comparison of 2-dimensional and 3-dimensional power-Doppler imaging in complex adnexal masses for the prediction of ovarian cancer. Am J Obstet Gynecol 2005;192(3):807-812.

S9. Barrea C, Alkazaleh F, Ryan G, et al. Prenatal cardiovascular manifestations in the twin-to-twin transfusion syndrome recipients and the impact of therapeutic amnioreduction. Am J Obstet Gynecol 2005;192(3):892-902.

S10. Rust OA, Atlas RO, Kimmel S, Roberts WE, Hess LW. Does the presence of a funnel increase the risk of adverse perinatal outcome in a patient with a short cervix? Am J Obstet Gynecol 2005;192(4):1060-1066.

S11. Orlandi F, Rossi C, Orlandi E, Jakil MC, Hallahan TW, Macri VJ, Krantz DA. First-trimester screening for trisomy-21 using a simplified method to assess the presence or absence of the fetal nasal bone. Am J Obstet Gynecol 2005;192(4):1107-1111.

S12. Abramowicz JS, Rana S, Abramowicz S. Fetal cheek-to-cheek diameter in the prediction of mode of delivery. Am J Obstet Gynecol 2005;192(4):1205-1211.

S13. Fishman DA, Cohen L, Blank SV, et al. The role of ultrasound evaluation in the detection of early-stage epithelial ovarian cancer. Am J Obstet Gynecol 2005;192(4):1214-1221.

S14. Hammoud AO, Hammoud I, Bujold E, Gonik B, Diamond MP, Johnson SC. The role of sonographic endometrial patterns and endometrial thickness in the differential diagnosis of ectopic pregnancy. Am J Obstet Gynecol 2005;192(5):1370-1375.

S15. Gutman RE, Pannu HK, Cundiff GW, Melick CF, Siddique SA, Handa VL. Anatomic relationship between the vaginal apex and the bony architecture of the pelvis: a magnetic resonance imaging evaluation. Am J Obstet Gynecol 2005;192(5):1544-1548.

S16. Letterie GS. Three-dimensional ultrasound-guided embryo transfer: a preliminary study. Am J Obstet Gynecol 2005;192(6):1983-1987.

S17. Gjelland K, Ekerhovd E, Granberg S. Transvaginal ultrasound-guided aspiration for treatment of tubo-ovarian abscess: a study of 302 cases. Am J Obstet Gynecol 2005;193(4):1323-1330.

S18. House M, O'Callaghan M, Bahrami S, et al. Magnetic resonance imaging of the cervix during pregnancy: effect of gestational age and prior vaginal birth. Am J Obstet Gynecol 2005;193(4):1554-1560.

S19. Bronshtein M, Blazer S, Zalel Y, Zimmer EZ. Ultrasonographic diagnosis of glossoptosis in fetuses with Pierre Robin sequence in early and mid pregnancy. Am J Obstet Gynecol 2005;193(4):1561-1564.

S20. Boreham MK, Zaretsky MV, Corton MM, Alexander JM, McIntire DD, Twickler DM. Appearance of the levator ani muscle in pregnancy as assessed by 3-D MRI. Am J Obstet Gynecol 2005;193(6):2159-2164.

S21. Zahalka N, Sadan O, Malinger G, Liberati M, Boaz M, Glezerman M, Rotmensch S. Comparison of transvaginal sonography with digital examination and transabdominal sonography for the determination of fetal head position in the second stage of labor. Am J Obstet Gynecol 2005;193(2):381-386.

S22. Predanic M, Perni SC, Chasen ST, Baergen RN, Chervenak FA. Ultrasound evaluation of abnormal umbilical cord coiling in second trimester of gestation in association with adverse pregnancy outcome. Am J Obstet Gynecol 2005;193(2):387-394.

S23. Wataganara T, Metzenbauer M, Peter I, Johnson KL, Bianchi DW. Placental volume, as measured by 3-dimensional sonography and levels of maternal plasma cell-free fetal DNA. Am J Obstet Gynecol 2005;193(2):496-500.

S24. McAuliffe FM, Trines J, Nield LE, Chitayat D, Jaeggi E, Hornberger LK. Early fetal echocardiography--a reliable prenatal diagnosis tool. Am J Obstet Gynecol 2005;193(3 Pt 2):1253-1259.

S25. McAuliffe FM, Fong KW, Toi A, Chitayat D, Keating S, Johnson JA. Ultrasound detection of fetal anomalies in conjunction with first-trimester nuchal translucency screening: a feasibility study. Am J Obstet Gynecol 2005;193(3 Pt 2):1260-1265.
S26. Coco C, Jeanty P. Isolated fetal pyelectasis and chromosomal abnormalities. Am J Obstet Gynecol 2005;193(3 Pt 1):732-738.
S27. Buhimschi CS, Buhimschi IA, Norwitz ER, et al. Sonographic myometrial thickness predicts the latency interval of women with preterm premature rupture of the membranes and oligohydramnios. Am J Obstet Gynecol 2005;193(3 Pt 1):762-770.
S28. Nakano Y, Wong JC, de Jong PA, et al. The prediction of small airway dimensions using computed tomography. Am J Respir Crit Care Med 2005;171(2):142-146.
S29. Herth FJ, Lunn W, Eberhardt R, Becker HD, Ernst A. Transbronchial versus transesophageal ultrasound-guided aspiration of enlarged mediastinal lymph nodes. Am J Respir Crit Care Med 2005;171(10):1164-1167.
S30. O'Donoghue FJ, Briellmann RS, Rochford PD, et al. Cerebral structural changes in severe obstructive sleep apnea. Am J Respir Crit Care Med 2005;171(10):1185-1190.
S31. Wilkins MR, Paul GA, Strange JW, et al. Sildenafil versus Endothelin Receptor Antagonist for Pulmonary Hypertension (SERAPH) study. Am J Respir Crit Care Med 2005;171(11):1292-1297.
S32. Arens R, Sin S, McDonough JM, et al. Changes in upper airway size during tidal breathing in children with obstructive sleep apnea syndrome. Am J Respir Crit Care Med 2005;171(11):1298-1304.
S33. Usmani OS, Biddiscombe MF, Barnes PJ. Regional lung deposition and bronchodilator response as a function of beta2-agonist particle size. Am J Respir Crit Care Med 2005;172(12):1497-1504.

S34. Goldbart AD, Goldman JL, Veling MC, Gozal D. Leukotriene modifier therapy for mild sleep-disordered breathing in children. Am J Respir Crit Care Med 2005;172(3):364-370.

S35. Lynch DA, David Godwin J, Safrin S, et al. High-resolution computed tomography in idiopathic pulmonary fibrosis: diagnosis and prognosis. Am J Respir Crit Care Med 2005;172(4):488-493.

S36. Wiegand LC, Warfield SK, Levitt JJ, et al. An in vivo MRI study of prefrontal cortical complexity in first-episode psychosis. Am J Psychiatry 2005;162(1):65-70.

S37. Kerns JG, Cohen JD, MacDonald AW 3rd, Johnson MK, Stenger VA, Aizenstein H, Carter CS. Decreased conflict- and error-related activity in the anterior cingulate cortex in subjects with schizophrenia. Am J Psychiatry 2005;162(10):1833-1839.

S38. Ragland JD, Gur RC, Valdez JN, et al. Levels-of-processing effect on frontotemporal function in schizophrenia during word encoding and recognition. Am J Psychiatry 2005;162(10):1840-1848.

S39. Tan HY, Choo WC, Fones CS, Chee MW. fMRI study of maintenance and manipulation processes within working memory in first-episode schizophrenia. Am J Psychiatry 2005;162(10):1849-1858.

S40. Armony JL, Corbo V, Clément MH, Brunet A. Amygdala response in patients with acute PTSD to masked and unmasked emotional facial expressions. Am J Psychiatry 2005;162(10):1961-1963.

S41. Gould RL, Brown RG, Owen AM, Bullmore ET, Williams SC, Howard RJ. Functional neuroanatomy of successful paired associate learning in Alzheimer's disease. Am J Psychiatry 2005;162(11):2049-2060.

S42. Freo U, Ricciardi E, Pietrini P, Schapiro MB, Rapoport SI, Furey ML. Pharmacological modulation of prefrontal cortical activity during a working memory task in young and older humans: a PET study with physostigmine. Am J Psychiatry 2005;162(11):2061-2070.

S43. Sassi RB, Stanley JA, Axelson D, et al. Reduced NAA levels in the dorsolateral prefrontal cortex of young bipolar patients. Am J Psychiatry 2005;162(11):2109-2115.

S44. Tamagaki C, Sedvall GC, Jönsson EG, Okugawa G, Hall H, Pauli S, Agartz I. Altered white matter/gray matter proportions in the striatum of patients with schizophrenia: a volumetric MRI study. Am J Psychiatry 2005;162(12):2315-2321.

S45. Snitz BE, MacDonald A 3rd, Cohen JD, Cho RY, Becker T, Carter CS. Lateral and medial hypofrontality in first-episode schizophrenia: functional activity in a medication-naive state and effects of short-term atypical antipsychotic treatment. Am J Psychiatry 2005;162(12):2322-2329.

S46. Narendran R, Frankle WG, Keefe R, et al. Altered prefrontal dopaminergic function in chronic recreational ketamine users. Am J Psychiatry 2005;162(12):2352-2359.

S47. Chang L, Ernst T, Speck O, Grob CS. Additive effects of HIV and chronic methamphetamine use on brain metabolite abnormalities. Am J Psychiatry 2005;162(2):361-369.

S48. Tregellas JR, Tanabe JL, Martin LF, Freedman R. FMRI of response to nicotine during a smooth pursuit eye movement task in schizophrenia. Am J Psychiatry 2005;162(2):391-393.

S49. MacDonald AW 3rd, Carter CS, Kerns JG, et al. Specificity of prefrontal dysfunction and context processing deficits to schizophrenia in never-medicated patients with first-episode psychosis. Am J Psychiatry 2005;162(3):475-484.

S50. Fu CH, Suckling J, Williams SC, Andrew CM, Vythelingum GN, McGuire PK. Effects of psychotic state and task demand on prefrontal function in schizophrenia: an fMRI study of overt verbal fluency. Am J Psychiatry 2005;162(3):485-494.

S51. Zubieta JK, Heitzeg MM, Xu Y, Koeppe RA, Ni L, Guthrie S, Domino EF. Regional cerebral blood flow responses to smoking in tobacco smokers after overnight abstinence. Am J Psychiatry 2005;162(3):567-577.

S52. Onitsuka T, Nestor PG, Gurrera RJ, et al. Association between reduced extraversion and right posterior fusiform gyrus gray matter reduction in chronic schizophrenia. Am J Psychiatry 2005;162(3):599-601.

S53. Szeszko PR, Ardekani BA, Ashtari M, et al. White matter abnormalities in first-episode schizophrenia or schizoaffective disorder: a diffusion tensor imaging study. Am J Psychiatry 2005;162(3):602-605.

S54. Modrego PJ, Fayed N, Pina MA. Conversion from mild cognitive impairment to probable Alzheimer's disease predicted by brain magnetic resonance spectroscopy. Am J Psychiatry 2005;162(4):667-675.

S55. Frankle WG, Lombardo I, New AS, et al. Brain serotonin transporter distribution in subjects with impulsive aggressivity: a positron emission study with [11C]McN 5652. Am J Psychiatry 2005;162(5):915-923.

S56. Lehrer DS, Christian BT, Mantil J, et al. Thalamic and prefrontal FDG uptake in never medicated patients with schizophrenia. Am J Psychiatry 2005;162(5):931-938.

S57. Bremner JD, Fani N, Ashraf A, et al. Functional brain imaging alterations in acne patients treated with isotretinoin. Am J Psychiatry 2005;162(5):983-991.

S58. Rubia K, Smith AB, Brammer MJ, Toone B, Taylor E. Abnormal brain activation during inhibition and error detection in medication-naive adolescents with ADHD. Am J Psychiatry 2005;162(6):1067-1075.

S59. Frazier JA, Chiu S, Breeze JL, et al. Structural brain magnetic resonance imaging of limbic and thalamic volumes in pediatric bipolar disorder. Am J Psychiatry 2005;162(7):1256-1265.

S60. Jernigan TL, Gamst AC, Archibald SL, et al. Effects of methamphetamine dependence and HIV infection on cerebral morphology. Am J Psychiatry 2005;162(8):1461-1472.

S61. Heinz A, Siessmeier T, Wrase J, et al. Correlation of alcohol craving with striatal dopamine synthesis capacity and D2/3 receptor availability: a combined [18F]DOPA and [18F]DMFP PET study in detoxified alcoholic patients. Am J Psychiatry 2005;162(8):1515-1520.
S62. Vaidya CJ, Bunge SA, Dudukovic NM, Zalecki CA, Elliott GR, Gabrieli JD. Altered neural substrates of cognitive control in childhood ADHD: evidence from functional magnetic resonance imaging. Am J Psychiatry 2005;162(9):1605-1613.
S63. Kaur S, Sassi RB, Axelson D, et al. Cingulate cortex anatomical abnormalities in children and adolescents with bipolar disorder. Am J Psychiatry 2005;162(9):1637-1643.
S64. Strakowski SM, Adler CM, Holland SK, Mills NP, DelBello MP, Eliassen JC. Abnormal FMRI brain activation in euthymic bipolar disorder patients during a counting Stroop interference task. Am J Psychiatry 2005;162(9):1697-1705.
S65. Coryell W, Nopoulos P, Drevets W, Wilson T, Andreasen NC. Subgenual prefrontal cortex volumes in major depressive disorder and schizophrenia: diagnostic specificity and prognostic implications. Am J Psychiatry 2005;162(9):1706-1712.
S66. Eckstein F, Charles HC, Buck RJ, et al. Accuracy and precision of quantitative assessment of cartilage morphology by magnetic resonance imaging at 3.0T. Arthritis Rheum 2005;52(10):3132-3136.
S67. Amin S, LaValley MP, Guermazi A, et al. The relationship between cartilage loss on magnetic resonance imaging and radiographic progression in men and women with knee osteoarthritis. Arthritis Rheum 2005;52(10):3152-3159.
S68. Loeuille D, Chary-Valckenaere I, Champigneulle J, et al. Macroscopic and microscopic features of synovial membrane inflammation in the osteoarthritic knee: correlating magnetic resonance imaging findings with disease severity. Arthritis Rheum 2005;52(11):3492-3501.

S69. Roos EM, Dahlberg L. Positive effects of moderate exercise on glycosaminoglycan content in knee cartilage: a four-month, randomized, controlled trial in patients at risk of osteoarthritis. Arthritis Rheum 2005;52(11):3507-3514.
S70. Williams A, Sharma L, McKenzie CA, Prasad PV, Burstein D. Delayed gadolinium-enhanced magnetic resonance imaging of cartilage in knee osteoarthritis: findings at different radiographic stages of disease and relationship to malalignment. Arthritis Rheum 2005;52(11):3528-3535.
S71. LaValley MP, McLaughlin S, Goggins J, Gale D, Nevitt MC, Felson DT. The lateral view radiograph for assessment of the tibiofemoral joint space in knee osteoarthritis: its reliability, sensitivity to change, and longitudinal validity. Arthritis Rheum 2005;52(11):3542-3547.
S72. Arabshahi B, Dewitt EM, Cahill AM, Kaye RD, Baskin KM, Towbin RB, Cron RQ. Utility of corticosteroid injection for temporomandibular arthritis in children with juvenile idiopathic arthritis. Arthritis Rheum 2005;52(11):3563-3569.
S73. Böttcher J, Pfeil A, Rosholm A, et al. Digital X-ray radiogrammetry combined with semiautomated analysis of joint space widths as a new diagnostic approach in rheumatoid arthritis: a cross-sectional and longitudinal study. Arthritis Rheum 2005;52(12):3850-3859.
S74. Haavardsholm EA, Ostergaard M, Ejbjerg BJ, Kvan NP, Uhlig TA, Lilleås FG, Kvien TK. Reliability and sensitivity to change of the OMERACT rheumatoid arthritis magnetic resonance imaging score in a multireader, longitudinal setting. Arthritis Rheum 2005;52(12):3860-3867.
S75. Kruithof E, De Rycke L, Roth J, et al. Immunomodulatory effects of etanercept on peripheral joint synovitis in the spondylarthropathies. Arthritis Rheum 2005;52(12):3898-3909.
S76. Ding C, Cicuttini F, Scott F, Boon C, Jones G. Association of prevalent and incident knee cartilage defects with loss of tibial and patellar cartilage: a longitudinal study. Arthritis Rheum 2005;52(12):3918-3927.

S77. Scheel AK, Hermann KG, Kahler E, et al. A novel ultrasonographic synovitis scoring system suitable for analyzing finger joint inflammation in rheumatoid arthritis. Arthritis Rheum 2005;52(3):733-743.
S78. McQueen F, Beckley V, Crabbe J, Robinson E, Yeoman S, Stewart N. Magnetic resonance imaging evidence of tendinopathy in early rheumatoid arthritis predicts tendon rupture at six years. Arthritis Rheum 2005;52(3):744-751.
S79. Hill CL, Seo GS, Gale D, Totterman S, Gale ME, Felson DT. Cruciate ligament integrity in osteoarthritis of the knee. Arthritis Rheum 2005;52(3):794-799.
S80. Zhai G, Cicuttini F, Srikanth V, Cooley H, Ding C, Jones G. Factors associated with hip cartilage volume measured by magnetic resonance imaging: the Tasmanian Older Adult Cohort Study. Arthritis Rheum 2005;52(4):1069-1076.
S81. Baraliakos X, Davis J, Tsuji W, Braun J. Magnetic resonance imaging examinations of the spine in patients with ankylosing spondylitis before and after therapy with the tumor necrosis factor alpha receptor fusion protein etanercept. Arthritis Rheum 2005;52(4):1216-1223.
S82. Conrozier T, Mathieu P, Piperno M, et al. Selection of knee radiographs for trials of structure-modifying drugs in patients with knee osteoarthritis: a prospective, longitudinal study of Lyon Schuss knee radiographs with the definition of adequate alignment of the medial tibial plateau. Arthritis Rheum 2005;52(5):1411-1417.
S83. Giesecke T, Gracely RH, Williams DA, Geisser ME, Petzke FW, Clauw DJ. The relationship between depression, clinical pain, and experimental pain in a chronic pain cohort. Arthritis Rheum 2005;52(5):1577-1584.
S84. Cicuttini F, Ding C, Wluka A, Davis S, Ebeling PR, Jones G. Association of cartilage defects with loss of knee cartilage in healthy, middle-age adults: a prospective study. Arthritis Rheum 2005;52(7):2033-2039.

S85. Benseler SM, deVeber G, Hawkins C, et al. Angiography-negative primary central nervous system vasculitis in children: a newly recognized inflammatory central nervous system disease. Arthritis Rheum 2005;52(7):2159-2167.
S86. Tan AL, Grainger AJ, Tanner SF, Shelley DM, Pease C, Emery P, McGonagle D. High-resolution magnetic resonance imaging for the assessment of hand osteoarthritis. Arthritis Rheum 2005;52(8):2355-2365.
S87. Bley TA, Weiben O, Uhl M, Vaith P, Schmidt D, Warnatz K, Langer M. Assessment of the cranial involvement pattern of giant cell arteritis with 3T magnetic resonance imaging. Arthritis Rheum 2005;52(8):2470-2477.
S88. Appenzeller S, Rondina JM, Li LM, Costallat LT, Cendes F. Cerebral and corpus callosum atrophy in systemic lupus erythematosus. Arthritis Rheum 2005;52(9):2783-2789.
S89. Lo GH, Hunter DJ, Zhang Y, et al. Bone marrow lesions in the knee are associated with increased local bone density. Arthritis Rheum 2005;52(9):2814-2821.
S90. Hofmann M, Wollert KC, Meyer GP, et al. Monitoring of bone marrow cell homing into the infarcted human myocardium. Circulation 2005;111(17):2198-202.
S91. Sousa JE, Costa MA, Abizaid A, et al. Four-year angiographic and intravascular ultrasound follow-up of patients treated with sirolimus-eluting stents. Circulation 2005;111(18):2326-2329.
S92. Satran A, Bart BA, Henry CR, Murad MB, Talukdar S, Satran D, Henry TD. Increased prevalence of coronary artery aneurysms among cocaine users. Circulation 2005;111(19):2424-2429.
S93. van Dockum WG, Beek AM, ten Cate FJ, et al. Early onset and progression of left ventricular remodeling after alcohol septal ablation in hypertrophic obstructive cardiomyopathy. Circulation 2005;111(19):2503-2508.
S94. Glaser R, Selzer F, Faxon DP, et al. Clinical progression of incidental, asymptomatic lesions discovered during culprit vessel coronary intervention. Circulation 2005;111(2):143-149.

S95. Maceira AM, Joshi J, Prasad SK, et al. Cardiovascular magnetic resonance in cardiac amyloidosis. Circulation 2005;111(2):186-193.
S96. Karch MR, Zrenner B, Deisenhofer I, et al. Freedom from atrial tachyarrhythmias after catheter ablation of atrial fibrillation: a randomized comparison between 2 current ablation strategies. Circulation 2005;111(22):2875-2880.
S97. Pietryga M, Brazert J, Wender-Ozegowska E, Biczysko R, Dubiel M, Gudmundsson S. Abnormal uterine Doppler is related to vasculopathy in pregestational diabetes mellitus. Circulation 2005;112(16):2496-2500.
S98. Dufouil C, Chalmers J, Coskun O, et al. Effects of blood pressure lowering on cerebral white matter hyperintensities in patients with stroke: the PROGRESS (Perindopril Protection Against Recurrent Stroke Study) Magnetic Resonance Imaging Substudy. Circulation 2005;112(11):1644-1650.
S99. Nesta F, Leyne M, Yosefy C, et al. New locus for autosomal dominant mitral valve prolapse on chromosome 13: clinical insights from genetic studies. Circulation 2005;112(13):2022-2030.
S100. Dilsizian V, Bateman TM, Bergmann SR, et al. Metabolic imaging with beta-methyl-p-[(123)I]-iodophenyl-pentadecanoic acid identifies ischemic memory after demand ischemia. Circulation 2005;112(14):2169-2174.
S101. Mollet NR, Cademartiri F, van Mieghem CA, et al. High-resolution spiral computed tomography coronary angiography in patients referred for diagnostic conventional coronary angiography. Circulation 2005;112(15):2318-2323.
S102. Sheehan R, Perloff JK, Fishbein MC, Gjertson D, Aberle DR. Pulmonary neovascularity: a distinctive radiographic finding in Eisenmenger syndrome. Circulation 2005;112(18):2778-2785.

S103. Nazarian S, Bluemke DA, Lardo AC, et al. Magnetic resonance assessment of the substrate for inducible ventricular tachycardia in nonischemic cardiomyopathy. Circulation 2005;112(18):2821-2825.
S104. Yotti R, Bermejo J, Antoranz JC, et al. A noninvasive method for assessing impaired diastolic suction in patients with dilated cardiomyopathy. Circulation 2005;112(19):2921-2929.
S105. Fries R, Shariat K, von Wilmowsky H, Böhm M. Sildenafil in the treatment of Raynaud's phenomenon resistant to vasodilatory therapy. Circulation 2005;112(19):2980-2985.
S106. Banki NM, Kopelnik A, Dae MW, et al. Acute neurocardiogenic injury after subarachnoid hemorrhage. Circulation 2005;112(21):3314-3319.
S107. Møller JE, Pellikka PA, Bernheim AM, Schaff HV, Rubin J, Connolly HM. Prognosis of carcinoid heart disease: analysis of 200 cases over two decades. Circulation 2005;112(21):3320-3327.
S108. Ector J, De Buck S, Adams J, Dymarkowski S, Bogaert J, Maes F, Heidbüchel H. Cardiac three-dimensional magnetic resonance imaging and fluoroscopy merging: a new approach for electroanatomic mapping to assist catheter ablation. Circulation 2005;112(24):3769-3776.
S109. Hanekom L, Jenkins C, Jeffries L, Case C, Mundy J, Hawley C, Marwick TH. Incremental value of strain rate analysis as an adjunct to wall-motion scoring for assessment of myocardial viability by dobutamine echocardiography: a follow-up study after revascularization. Circulation 2005;112(25):3892-3900.
S110. van der Lee C, ten Cate FJ, Geleijnse ML, et al. Percutaneous versus surgical treatment for patients with hypertrophic obstructive cardiomyopathy and enlarged anterior mitral valve leaflets. Circulation 2005;112(4):482-488.
S111. Rickers C, Wilke NM, Jerosch-Herold M, et al. Utility of cardiac magnetic resonance imaging in the diagnosis of hypertrophic cardiomyopathy. Circulation 2005;112(6):855-861.

S112. Bartunek J, Vanderheyden M, Vandekerckhove B, et al. Intracoronary injection of CD133-positive enriched bone marrow progenitor cells promotes cardiac recovery after recent myocardial infarction: feasibility and safety. Circulation 2005;112(9 Suppl):I178-I183.
S113. Koschyk DH, Nienaber CA, Knap M, et al. How to guide stent-graft implantation in type B aortic dissection? Comparison of angiography, transesophageal echocardiography, and intravascular ultrasound. Circulation 2005;112(9 Suppl):I260-I264.
S114. Glineur D, Noirhomme P, Reisch J, El Khoury G, Astarci P, Hanet C. Resistance to flow of arterial Y-grafts 6 months after coronary artery bypass surgery. Circulation. 2005 Aug 30;112(9 Suppl):I281-I285.
S115. Boekstegers P, Raake P, Hinkel R, et al. Hemodynamic and vascular effects of ventricular sourcing by stent-based ventricle to coronary artery bypass in patients with multivessel disease undergoing coronary artery bypass surgery. Circulation 2005;112(9 Suppl):I304-I310.
S116. Williams IA, Quaegebeur JM, Hsu DT, et al. Ross procedure in infants and toddlers followed into childhood. Circulation 2005;112(9 Suppl):I390-I395.
S117. Zhu F, Otsuji Y, Yotsumoto G, et al. Mechanism of persistent ischemic mitral regurgitation after annuloplasty: importance of augmented posterior mitral leaflet tethering. Circulation 2005;112(9 Suppl):I396-I401.
S118. Kaji S, Nasu M, Yamamuro A, et al. Annular geometry in patients with chronic ischemic mitral regurgitation: three-dimensional magnetic resonance imaging study. Circulation 2005;112(9 Suppl):I409-I414.
S119. Westenberg JJ, van der Geest RJ, Lamb HJ, et al. MRI to evaluate left atrial and ventricular reverse remodeling after restrictive mitral annuloplasty in dilated cardiomyopathy. Circulation 2005;112(9 Suppl):I437-I442.

S120. Vandenbergh J, Dupont P, Fischler B, Bormans G, Persoons P, Janssens J, Tack J. Regional brain activation during proximal stomach distention in humans: A positron emission tomography study. Gastroenterology 2005;128(3):564-573.

S121. Salvioli B, Serra J, Azpiroz F, Lorenzo C, Aguade S, Castell J, Malagelada JR. Origin of gas retention and symptoms in patients with bloating. Gastroenterology 2005;128(3):574-579.

S122. Jung HY, Puckett JL, Bhalla V, Rojas-Feria M, Bhargava V, Liu J, Mittal RK. Asynchrony between the circular and the longitudinal muscle contraction in patients with nutcracker esophagus. Gastroenterology 2005;128(5):1179-1186.

S123. Bharucha AE, Fletcher JG, Seide B, Riederer SJ, Zinsmeister AR. Phenotypic variation in functional disorders of defecation. Gastroenterology 2005;128(5):1199-1210.

S124. Peñuelas I, Mazzolini G, Boán JF, et al. Positron emission tomography imaging of adenoviral-mediated transgene expression in liver cancer patients. Gastroenterology 2005;128(7):1787-1795.

S125. Yágüez L, Coen S, Gregory LJ, et al. Brain response to visceral aversive conditioning: a functional magnetic resonance imaging study. Gastroenterology 2005;128(7):1819-1829.

S126. Hoffmann MH, Shi H, Schmitz BL, et al. Noninvasive coronary angiography with multislice computed tomography. JAMA 2005;293(20):2471-2478.

S127. Geng E, Kreiswirth B, Burzynski J, Schluger NW. Clinical and radiographic correlates of primary and reactivation tuberculosis: a molecular epidemiology study. JAMA 2005;293(22):2740-2745.

S128. Schiavi F, Boedeker CC, Bausch B, et al. Predictors and prevalence of paraganglioma syndrome associated with mutations of the SDHC gene. JAMA 2005;294(16):2057-2063.

S129. Rockey DC, Paulson E, Niedzwiecki D, et al. Analysis of air contrast barium enema, computed tomographic colonography, and colonoscopy: prospective comparison. Lancet 2005;365(9456):305-3011.

S130. Skilton MR, Evans N, Griffiths KA, Harmer JA, Celermajer DS. Aortic wall thickness in newborns with intrauterine growth restriction. Lancet 2005;365(9469):1484-1486.

S131. Mayhew PM, Thomas CD, Clement JG, et al. Relation between age, femoral neck cortical stability, and hip fracture risk. Lancet 2005;366(9480):129-135.

S132. Greenman RL, Panasyuk S, Wang X, et al. Early changes in the skin microcirculation and muscle metabolism of the diabetic foot. Lancet 2005;366(9498):1711-1717.

S133. Escolar ML, Poe MD, Provenzale JM, et al. Transplantation of umbilical-cord blood in babies with infantile Krabbe's disease. N Engl J Med 2005;352(20):2069-2081.

S134. Escolar ML, Poe MD, Provenzale JM, et al. Transplantation of umbilical-cord blood in babies with infantile Krabbe's disease. N Engl J Med 2005;352(20):2069-2081.

S135. Arad M, Maron BJ, Gorham JM, et al. Glycogen storage diseases presenting as hypertrophic cardiomyopathy. N Engl J Med 2005;352(4):362-372.

S136. Kaminski MS, Tuck M, Estes J, et al. 131I-tositumomab therapy as initial treatment for follicular lymphoma. N Engl J Med 2005;352(5):441-449.

S137. Wittstein IS, Thiemann DR, Lima JA, et al. Neurohumoral features of myocardial stunning due to sudden emotional stress. N Engl J Med 2005;352(6):539-548.

S138. Dragun D, Müller DN, Bräsen JH, et al. Angiotensin II type 1-receptor activating antibodies in renal-allograft rejection. N Engl J Med 2005;352(6):558-569.

S139. Drzezga A, Riemenschneider M, Strassner B, et al. Cerebral glucose metabolism in patients with AD and different APOE genotypes. Neurology 2005;64(1):102-107.

S140. Raeder MB, Helland CA, Hugdahl K, Wester K. Arachnoid cysts cause cognitive deficits that improve after surgery. Neurology 2005;64(1):160-162.

S141. Mosconi L, Tsui WH, De Santi S, et al. Reduced hippocampal metabolism in MCI and AD: automated FDG-PET image analysis. Neurology 2005;64(11):1860-1867.

S142. Josephs KA, Ahlskog JE, Klos KJ, Kumar N, Fealey RD, Trenerry MR, Cowl CT. Neurologic manifestations in welders with pallidal MRI T1 hyperintensity. Neurology 2005;64(12):2033-2039.

S143. Oba H, Yagishita A, Terada H, et al. New and reliable MRI diagnosis for progressive supranuclear palsy. Neurology 2005;64(12):2050-2055.

S144. Nagano-Saito A, Washimi Y, Arahata Y, et al. Cerebral atrophy and its relation to cognitive impairment in Parkinson disease. Neurology 2005;64(2):224-229.

S145. Rambold H, Sander T, Neumann G, Helmchen C. Palsy of "fast" and "slow" vergence by pontine lesions. Neurology 2005;64(2):338-340.

S146. Harno H, Heikkinen S, Kaunisto MA, et al. Decreased cerebellar total creatine in episodic ataxia type 2: a 1H MRS study. Neurology 2005;64(3):542-544.

S147. Udd B, Vihola A, Sarparanta J, Richard I, Hackman P. Titinopathies and extension of the M-line mutation phenotype beyond distal myopathy and LGMD2J. Neurology 2005;64(4):636-642.

S148. Riecker A, Mathiak K, Wildgruber D, Erb M, Hertrich I, Grodd W, Ackermann H. fMRI reveals two distinct cerebral networks subserving speech motor control. Neurology 2005;64(4):700-706.

S149. Thömke F, Marx JJ, Iannetti GD, et al. A topodiagnostic investigation on body lateropulsion in medullary infarcts. Neurology 2005;64(4):716-718.

S150. Jeong Y, Park KC, Cho SS, et al. Pattern of glucose hypometabolism in frontotemporal dementia with motor neuron disease. Neurology 2005;64(4):734-736.

S151. Karnath HO, Johannsen L, Broetz D, Küker W. Posterior thalamic hemorrhage induces "pusher syndrome". Neurology 2005;64(6):1014-1019.

S152. Schreckenberger M, Siessmeier T, Viertmann A, et al. The unpleasantness of tonic pain is encoded by the insular cortex. Neurology 2005;64(7):1175-1183.

S153. Kim JS, Kim J. Pure midbrain infarction: clinical, radiologic, and pathophysiologic findings. Neurology 2005;64(7):1227-1232.

S154. Kobayashi E, Bagshaw AP, Jansen A, Andermann F, Andermann E, Gotman J, Dubeau F. Intrinsic epileptogenicity in polymicrogyric cortex suggested by EEG-fMRI BOLD responses. Neurology 2005;64(7):1263-1266.

S155. Wojner-Alexander AW, Garami Z, Chernyshev OY, Alexandrov AV. Heads down: flat positioning improves blood flow velocity in acute ischemic stroke. Neurology 2005;64(8):1354-1357.

S156. Seeley WW, Bauer AM, Miller BL, Gorno-Tempini ML, Kramer JH, Weiner M, Rosen HJ. The natural history of temporal variant frontotemporal dementia. Neurology 2005;64(8):1384-1390.

S157. Stoub TR, Bulgakova M, Leurgans S, Bennett DA, Fleischman D, Turner DA, deToledo-Morrell L. MRI predictors of risk of incident Alzheimer disease: a longitudinal study. Neurology 2005;64(9):1520-1524.

S158. Meissner B, Westner IM, Kallenberg K, et al. Sporadic Creutzfeldt-Jakob disease: clinical and diagnostic characteristics of the rare VV1 type. Neurology 2005;65(10):1544-1550.

S159. O'Sullivan M, Barrick TR, Morris RG, Clark CA, Markus HS. Damage within a network of white matter regions underlies executive dysfunction in CADASIL. Neurology 2005;65(10):1584-1590.

S160. Rusu V, Chassoux F, Landré E, et al. Dystonic posturing in seizures of mesial temporal origin: electroclinical and metabolic patterns. Neurology 2005;65(10):1612-1619.

S161. Werring DJ, Coward LJ, Losseff NA, Jäger HR, Brown MM. Cerebral microbleeds are common in ischemic stroke but rare in TIA. Neurology 2005;65(12):1914-1918.

S162. Tedeschi G, Lavorgna L, Russo P, et al. Brain atrophy and lesion load in a large population of patients with multiple sclerosis. Neurology 2005;65(2):280-285.

S163. Wang HC, Chang WN, Lui CC, et al. The prognosis of hearing impairment complicating HIV-negative cryptococcal meningitis. Neurology 2005;65(2):320-322.
S164. Lanczik O, Szabo K, Hennerici M, Gass A. Multiparametric MRI and ultrasound findings in patients with internal carotid artery dissection. Neurology 2005;65(3):469-471.
S165. Knake S, Triantafyllou C, Wald LL, et al. 3T phased array MRI improves the presurgical evaluation in focal epilepsies: a prospective study. Neurology 2005;65(7):1026-1031.
S166. Lin JJ, Salamon N, Dutton RA, et al. Three-dimensional preoperative maps of hippocampal atrophy predict surgical outcomes in temporal lobe epilepsy. Neurology 2005;65(7):1094-1097.
S167. Misu T, Fujihara K, Nakashima I, Sato S, Itoyama Y. Intractable hiccup and nausea with periaqueductal lesions in neuromyelitis optica. Neurology 2005;65(9):1479-1482.
S168. Schmidt-Wilcke T, Leinisch E, Straube A, et al. Gray matter decrease in patients with chronic tension type headache. Neurology 2005;65(9):1483-1486.
S169. Chen L, Hsiao AL, Moore CL, Dziura JD, Santucci KA. Utility of bedside bladder ultrasound before urethral catheterization in young children. Pediatrics 2005;115(1):108-111.
S170. Valente AM, Jain R, Scheurer M, et al. Frequency of infective endocarditis among infants and children with Staphylococcus aureus bacteremia. Pediatrics 2005;115(1):e15-e19.
S171. Inder TE, Warfield SK, Wang H, Hüppi PS, Volpe JJ. Abnormal cerebral structure is present at term in premature infants. Pediatrics 2005;115(2):286-294.
S172. Boardman JP, Ganesan V, Rutherford MA, Saunders DE, Mercuri E, Cowan F. Magnetic resonance image correlates of hemiparesis after neonatal and childhood middle cerebral artery stroke. Pediatrics 2005;115(2):321-326.
S173. Fattal-Valevski A, Kesler A, Sela BA, et al. Outbreak of life-threatening thiamine deficiency in infants in Israel caused by a defective soy-based formula. Pediatrics 2005;115(2):e233-e238.

S174. Limperopoulos C, Soul JS, Gauvreau K, et al. Late gestation cerebellar growth is rapid and impeded by premature birth. Pediatrics 2005;115(3):688-695.
S175. van de Putte EM, Uiterwaal CS, Bots ML, Kuis W, Kimpen JL, Engelbert RH. Is chronic fatigue syndrome a connective tissue disorder? A cross-sectional study in adolescents. Pediatrics 2005;115(4):e415-e422.
S176. Lodygensky GA, Rademaker K, Zimine S, et al. Structural and functional brain development after hydrocortisone treatment for neonatal chronic lung disease. Pediatrics 2005;116(1):1-7.
S177. Khurana DS, Melvin JJ, Kothare SV, et al. Acute disseminated encephalomyelitis in children: discordant neurologic and neuroimaging abnormalities and response to plasmapheresis. Pediatrics 2005;116(2):431-436.
S178. Limperopoulos C, Benson CB, Bassan H, et al. Cerebellar hemorrhage in the preterm infant: ultrasonographic findings and risk factors. Pediatrics 2005;116(3):717-724.
S179. Adame N, Hedlund G, Byington CL. Sinogenic intracranial empyema in children. Pediatrics 2005;116(3):e461-e467.
S180. Rutherford MA, Azzopardi D, Whitelaw A, Cowan F, Renowden S, Edwards AD, Thoresen M. Mild hypothermia and the distribution of cerebral lesions in neonates with hypoxic-ischemic encephalopathy. Pediatrics 2005;116(4):1001-1006.
S181. Limperopoulos C, Soul JS, Haidar H, et al. Impaired trophic interactions between the cerebellum and the cerebrum among preterm infants. Pediatrics 2005;116(4):844-850.
S182. Jea A, Smith ER, Robertson R, Scott RM. Moyamoya syndrome associated with Down syndrome: outcome after surgical revascularization. Pediatrics 2005;116(5):e694-e701.
S183. Hermoye L, Laamari-Azjal I, Cao Z, Annet L, Lerut J, Dawant BM, Van Beers BE. Liver segmentation in living liver transplant donors: comparison of semiautomatic and manual methods. Radiology 2005;234(1):171-178.

S184. He J, Inglese M, Li BS, Babb JS, Grossman RI, Gonen O. Relapsing-remitting multiple sclerosis: metabolic abnormality in nonenhancing lesions and normal-appearing white matter at MR imaging: initial experience. Radiology 2005;234(1):211-217.

S185. Iannaccone R, Laghi A, Catalano C, et al. Hepatocellular carcinoma: role of unenhanced and delayed phase multi-detector row helical CT in patients with cirrhosis. Radiology 2005;234(2):460-467.

S186. Kuhl CK, Textor J, Gieseke J, von Falkenhausen M, Gernert S, Urbach H, Schild HH. Acute and subacute ischemic stroke at high-field-strength (3.0-T) diffusion-weighted MR imaging: intraindividual comparative study. Radiology 2005;234(2):509-516.

S187. Kim SH, Lee JM, Kim JH, et al. Appropriateness of a donor liver with respect to macrosteatosis: application of artificial neural networks to US images--initial experience. Radiology 2005;234(3):793-803.

S188. Hoenig K, Kuhl CK, Scheef L. Functional 3.0-T MR assessment of higher cognitive function: are there advantages over 1.5-T imaging? Radiology 2005;234(3):860-868.

S189. Hagiwara A, Fukushima H, Murata A, Matsuda H, Shimazaki S. Blunt splenic injury: usefulness of transcatheter arterial embolization in patients with a transient response to fluid resuscitation. Radiology 2005;235(1):57-64.

S190. Metser U, Haider MA, Dill-Macky M, Atri M, Lockwood G, Minden M. Fungal liver infection in immunocompromised patients: depiction with multiphasic contrast-enhanced helical CT. Radiology 2005;235(1):97-105.

S191. Elkington AG, Gatehouse PD, Cannell TM, Moon JC, Prasad SK, Firmin DN, Pennell DJ. Comparison of hybrid echo-planar imaging and FLASH myocardial perfusion cardiovascular MR imaging. Radiology 2005;235(1):237-243.

S192. Memarsadeghi M, Heinz-Peer G, Helbich TH, Schaefer-Prokop C, Kramer G, Scharitzer M, Prokop M. Unenhanced multi-detector row CT in patients suspected of having urinary stone disease: effect of section width on diagnosis. Radiology 2005;235(2):530-536.

S193. Wu F, Wang ZB, Chen WZ, et al. Advanced hepatocellular carcinoma: treatment with high-intensity focused ultrasound ablation combined with transcatheter arterial embolization. Radiology 2005;235(2):659-667.

S194. Khalil HI, Patterson SA, Panicek DM. Hepatic lesions deemed too small to characterize at CT: prevalence and importance in women with breast cancer. Radiology 2005;235(3):872-878.

S195. Wu C, Zhang J, Ladner CJ, Babb JS, Lamparello PJ, Krinsky GA. Subclavian steal syndrome: diagnosis with perfusion metrics from contrast-enhanced MR angiographic bolus-timing examination--initial experience. Radiology 2005;235(3):927-933.

S196. Zimmerman SL, Levine MS, Rubesin SE, Mitre MC, Furth EE, Laufer I, Katzka DA. Idiopathic eosinophilic esophagitis in adults: the ringed esophagus. Radiology 2005;236(1):159-165.

S197. van den Dool SW, Wasser MN, de Fijter JW, Hoekstra J, van der Geest RJ. Functional renal volume: quantitative analysis at gadolinium-enhanced MR angiography--feasibility study in healthy potential kidney donors. Radiology 2005;236(1):189-195.

S198. Anzalone N, Scomazzoni F, Castellano R, et al. Carotid artery stenosis: intraindividual correlations of 3D time-of-flight MR angiography, contrast-enhanced MR angiography, conventional DSA, and rotational angiography for detection and grading. Radiology 2005;236(1):204-213.

S199. Tack D, De Maertelaer V, Petit W, Scillia P, Muller P, Suess C, Gevenois PA. Multi-detector row CT pulmonary angiography: comparison of standard-dose and simulated low-dose techniques. Radiology 2005;236(1):318-325.

S200. Birdwell RL, Bandodkar P, Ikeda DM. Computer-aided detection with screening mammography in a university hospital setting. Radiology 2005;236(2):451-457.

S201. Meisamy S, Bolan PJ, Baker EH, et al. Adding in vivo quantitative 1H MR spectroscopy to improve diagnostic accuracy of breast MR imaging: preliminary results of observer performance study at 4.0 T. Radiology 2005;236(2):465-475.

S202. Silverman SG, Tuncali K, vanSonnenberg E, Morrison PR, Shankar S, Ramaiya N, Richie JP. Renal tumors: MR imaging-guided percutaneous cryotherapy--initial experience in 23 patients. Radiology 2005;236(2):716-724.

S203. Shemesh J, Evron R, Koren-Morag N, et al. Coronary artery calcium measurement with multi-detector row CT and low radiation dose: comparison between 55 and 165 mAs. Radiology 2005;236(3):810-814.

S204. Fleischmann D, Rubin GD. Quantification of intravenously administered contrast medium transit through the peripheral arteries: implications for CT angiography. Radiology 2005;236(3):1076-1082.

S205. Zhu Q, Cronin EB, Currier AA, Vine HS, Huang M, Chen N, Xu C. Benign versus malignant breast masses: optical differentiation with US-guided optical imaging reconstruction. Radiology 2005;237(1):57-66.

S206. Laissy JP, Hyafil F, Feldman LJ, Juliard JM, Schouman-Claeys E, Steg PG, Faraggi M. Differentiating acute myocardial infarction from myocarditis: diagnostic value of early- and delayed-perfusion cardiac MR imaging. Radiology 2005;237(1):75-82.

S207. Ward J, Robinson PJ, Guthrie JA, et al. Liver metastases in candidates for hepatic resection: comparison of helical CT and gadolinium- and SPIO-enhanced MR imaging. Radiology 2005;237(1):170-180.

S208. Rizzo SM, Kalra MK, Schmidt B, Raupach R, Maher MM, Blake MA, Saini S. CT images of abdomen and pelvis: effect of nonlinear three-dimensional optimized reconstruction algorithm on image quality and lesion characteristics. Radiology 2005;237(1):309-315.
S209. Chow LC, Napoli A, Klein MB, Chang J, Rubin GD. Vascular mapping of the leg with multi-detector row CT angiography prior to free-flap transplantation. Radiology 2005;237(1):353-360.
S210. Strobel K, Hodler J, Meyer DC, Pfirrmann CW, Pirkl C, Zanetti M. Fatty atrophy of supraspinatus and infraspinatus muscles: accuracy of US. Radiology 2005;237(2):584-589.
S211. Sitek A, Sheiman RG. Small-bowel perfusion measurement: feasibility with single-compartment kinetic model applied to dynamic contrast-enhanced CT. Radiology 2005;237(2):670-674.
S212. Carlson SK, Felmlee JP, Bender CE, et al. CT fluoroscopy-guided biopsy of the lung or upper abdomen with a breath-hold monitoring and feedback system: a prospective randomized controlled clinical trial. Radiology 2005;237(2):701-708.
